# External Treatment of Varicose Veins

**Published:** 1874-05

**Authors:** 


EXTERNAL TREATMENT OF VARICOSE VEINS.—If Dr. Linon, of Verviers, is right in his reports of his treatment of varicose veins, many who suffer from them will thank him for his discovery, as it saves them the pain and danger of an operation. He says, in the *Tribune Medicale,* that he has for years treated such cases with success by swathing the leg in a flannel compress wet with a solution of chloride of iron in water, forty-five grains to the ounce, and then applying a roller flannel bandage over it firmly for twenty-four hours. This is to be repeated daily for a week or two weeks, when the patient is, or ought to be, well.—*Medical and Surgical Reporter.*

